# Effectiveness of antiretroviral therapy and development of drug resistance in HIV-1 infected patients in Mombasa, Kenya

**DOI:** 10.1186/1742-6405-6-12

**Published:** 2009-06-16

**Authors:** Kim Steegen, Stanley Luchters, Kenny Dauwe, Jacqueline Reynaerts, Kishor Mandaliya, Walter Jaoko, Jean Plum, Marleen Temmerman, Chris Verhofstede

**Affiliations:** 1 International Centre for Reproductive Health, Ghent University, Ghent, Belgium; 2 International Centre for Reproductive Health, Mombasa, Kenya; 3 Aids Reference Laboratory, Ghent University, Ghent, Belgium; 4 Coast Provincial General Hospital, Mombasa, Kenya; 5 Department of Medical Microbiology, University of Nairobi, Nairobi, Kenya; 6 Current address : Virco BVBA, Generaal De Wittelaan L11b3, 2800 Mechelen, Belgium

## Abstract

Access to antiretroviral therapy (ART) is increasing in resource-limited settings (RLS) and can successfully reduce HIV-related morbidity and mortality. However, virologic failure and development of viral drug resistance can result in reduced treatment options and disease progression. Additionally, transmission of resistant virus, and particularly multi-drug resistance, could become a public health concern. This study evaluated treatment success and development of ART drug resistance after short-term treatment among patients attending the Comprehensive HIV Care Centre (CCC) of Coast Province General Hospital, Mombasa, Kenya. One hundred and fifty HIV-infected individuals receiving ART were consecutively recruited to participate in the study. After determination of plasma viral load, patients with detectable viral load levels were subjected to genotypic drug resistance testing. At the time of sampling, 132 of the 150 participants were on ART for more than 6 months (median 21 months, IQR = 12–26). An efficient viral load reduction to below 50 copies/ml was observed in 113 (85.6%) of them. Of the 19 patients with a detectable viral load, sequencing of the protease (PR) and reverse transcriptase (RT) gene was successful in 16. Eleven (11) of these 16 patients were infected with a subtype A1 virus. Major PR mutations were absent, but mutations associated with drug resistance in RT were detected in 14 of the 16 patients (87.5%). High-level resistance against at least 2 drugs of the ART regimen was observed in 9/14 (64.3%). The 3TC mutation M184V and the NNRTI mutation K103N were most frequent but also the multi-drug resistance Q151M and the broad NRTI cross-resistance K65R were observed. The results of this study revealed a high rate of treatment success after short term ART in patients treated at a public provincial hospital in a RLS. Nevertheless, the observed high risk of accumulation of resistance mutations among patients failing treatment and the selection of multi-drug resistance mutations in some, remains of great concern for future treatment options and potential transmission to partners.

## Introduction

Recent data show an HIV prevalence in Kenya of 7.4%, resulting in 1.4 million Kenyans living with HIV [1]. An estimated 190,000 HIV-infected Kenyans receive ART, representing 44% of those in need of treatment [1]. At the Comprehensive HIV Care Centre (CCC) in Mombasa, the decision on when to start or switch treatment is based on clinical and immunological parameters [2,3]. Information on the rate of treatment failure and the development of drug resistance in public hospitals in RLS, where treatment decisions are guided by clinical parameters and CD4 count only, is limited. Virologic treatment failure and accompanying resistance are of concern with regard to the risk of disease progression and potential transmission of drug resistant virus to partners. The aim of this study was to assess, in a cross-sectional survey, the rate of viral suppression and drug resistance among individuals receiving ART.

## Materials and methods

From the patients attending the CCC in Coast Province General Hospital, Mombasa, a total of 150 consecutive patients, over 18 years of age and receiving ART, were asked to participate in this surveillance study by a trained adherence counsellor. Fifty (50) patients were recruited in April 2006, 100 patients were recruited in May 2007. Participants gave written informed consent, and refusal to participate did not influence the standard of care. Ethical approval was obtained from the Kenyatta National Hospital Ethics and Research Committee.

Using a structured questionnaire, basic socio-demographic and clinical data was obtained from each participant. ART adherence was measured by self-report and pill count over the last month and recorded as satisfactory (>95%) or unsatisfactory (<95%). Ten (10) ml of EDTA blood was collected for CD4 cell count (FACScount Becton & Dickinson Immunocytometry, Oxford, UK). The remainder of the blood was centrifuged to collect plasma, which was stored at -80°C until processing for viral load measurement and genotyping.

Plasma HIV RNA quantification was performed, using the Ultrasensitive Cobas Amplicor HIV-1 Monitor Test version 1.5 (Roche Diagnostics, Basel, Switzerland) with a lower detection limit of 50 copies/ml.

Extraction, amplification and genotyping of HIV RNA was performed by nested RT-PCR and an in-house sequencing assay as described elsewhere [4]. The interpretations of genotyping data and subtyping were performed using Smartgene™ HIV software packages (Integrated Database Network System, Smartgene, Zug, Switzerland). Selection of drug resistance mutations was based on the recent update of the IAS-USA list [5]. Sequences were submitted to Genbank [Genbank EU872121–EU872135 and 878548 for PR and EU872136–EU872150 and 878549 for RT].

All statistical analyses were performed using SPSS 15.0 (SPSS, Illinois, USA).

## Results

The median age of the participants was 37 years (IQR = 32–43) with 69% being women (Table S1; Additional File 1). The majority of participants (67%) were in WHO clinical stage 3 or 4 [6]. Baseline CD4 count was available for 146 participants with a median of 112 cells/mm^3 ^(IQR = 63–184). A combination of d4T+3TC+NVP was the most commonly prescribed first-line regimen (n = 79), followed by d4T+3TC+EFV (n = 63), AZT+3TC+NVP (n = 5), AZT+3TC+EFV (n = 2), and d4T+ddI+EFV (n = 1). Patients receiving their first ART regimen had been treated for a median of 17 months (IQR = 10–24). In 16 patients treatment was changed after a median of 18 months (IQR = 13–26) by substituting one (n = 7), two (n = 5) or three (n = 4) drugs because of adverse events (n = 8), start of anti-tuberculosis treatment (n = 1), unavailability of drugs (n = 1), or immunological failure (n = 6). The 6 patients with immunological failure were switched to a LPV/r based regimen as recommended by the Kenyan national guidelines [2].

Eighteen of the 150 patients initiated ART less than 6 months (median 3.5 months, IQR = 2–6) before blood collection and were excluded from further analyses. For the remaining 132 patients, an undetectable viral load was seen in 110 (87.3%) of the 126 patients without treatment changes or with treatment changes for other reasons than immunological failure and in 3 of the 6 patients in whom the treatment was changed because of immunological failure. The median viral load of the 19 patients with ongoing viral replication was 3,060 copies/ml (IQR = 294–21,000). A detectable viral load was not significantly associated with the mean duration of ART (*P *= 0.42) or mean baseline CD4 (*P *= 0.18). A significantly higher mean CD4 count was seen in patients with an undetectable viral load (n = 113, mean = 344 cells/mm^3^) compared to those with a detectable viral load (n = 19, mean = 253 cells/mm^3^) (*P *= 0.03). However, the mean increase in CD4 from baseline level was not significantly different between the two groups (*P *= 0.33). Seventy-four patients (58.6%) reported to have satisfactory adherence, which was significantly associated with treatment success (*P *= 0.02).

Genotyping was attempted for all 19 participants with a detectable viral load and was successful for PR in 17 (89.5%) and for RT in 16 (84.2%). Amplification failures were due to low viral loads (79, 81 and 115 copies/ml). The subtype distribution for these 16 samples was as follows: subtype A1 (n = 11), CRF16_AD (n = 2), C (n = 1), D (n = 1) and a new recombinant of A1 and D (n = 1). No major PR resistance mutations were seen in any of the sequences, but a mean of 4 minor PR mutations were observed per sample (data not shown). Resistance mutations in RT were detected in 14 out of the 16 patients. Overall, the V184M was most commonly observed (n = 12), followed by K103N (n = 9). In one patient the multi-drug resistant Q151M mutation was seen and two patients carried virus with a K65R mutation, all three combined with V184M (Table S2; Additional File 2). In 10 patients a combination of V184M and at least one mutation conferring to NNRTI-class resistance was observed. Two patients who harboured wild type virus had a viral load of 533 and 1,380 copies/ml after 23 and 26 months of treatment respectively.

## Discussion

Intensive campaigns to improve availability of ART worldwide is paying off and most African countries are currently able to provide first-line ART regimens to a considerable number of HIV-1 infected individuals in need of treatment. Efforts to scale-up laboratory facilities for treatment monitoring in these patients however are running behind. A random sample survey is often the only way to assess treatment efficacy and the selection of drug resistance in treatment programs in Africa. The information obtained from these surveys is important for eventual future adaptation of treatment strategies. Moreover, resistance data obtained from these surveys will be crucial in evaluating the value of second-line regimens in Africa where only limited drugs are available.

In this study, 85.6% of the patients receiving ART ≥ 6 months had a VL<50 copies/ml. These figures are comparable to what has been published for other African regions [7-10]. The high treatment success rate seen in this study might be partly due to a bias because of possible selection of patients who attend the CCC more regularly. These patients could be more motivated and having a better treatment adherence. Although the total number of patients was small, a significant correlation between adherence and treatment success was demonstrated.

Despite the overall high efficacy of ART treatment regimens, a significant accumulation of resistance mutations was observed in patients with a detectable viral load at 6 months or more after treatment initiation. Though we cannot excluded that some of these mutations were already present at baseline, we assume that most of them were selected during treatment. As mutations can only be selected in the presence of the drug, their detection excludes the poor-intake of medication as the main reason for failure.

The combinations of AZT/d4T+3TC+EFV/NVP are extensively used as a first-line regimen in RLS [11]. Despite known toxicity of d4T, this drug is still commonly used in RLS as a component of the low-cost generic fixed dose combinations. 3TC, EFV and NVP have a low genetic barrier towards resistance and it is therefore not unexpected that, in accordance with the results of other studies, the 3TC mutation M184V and the NNRTI mutations K103N, 190G and 181C are frequently observed in case of treatment failure [8,12]. The high percentage (62.5%) of patients with a combination of M184V mutations and at least one NNRTI resistance associated mutation, as well as the selection of the broad NRTI cross-resistance mutations Q151M and K65R in 3 patients are worrying.

K65R mutations have previously been described among populations with similar subtypes [13]. However, this was mainly among patients receiving a TDF containing regimen which is known to induce the K65R mutation [14]. However, the selection of K65R under a d4T containing regimen seems to be more common among non-B subtypes as observed by others [15,16]. Despite the presence of the thymidine analogues AZT or d4T in most of the regimens, thymidine analogue mutations (TAMs) were infrequently observed.

Accumulation of mutations against drugs from different drug classes and/or the presence of broad cross-resistance mutations will jeopardize the effectiveness of the NRTI backbone of second-line regimens that often include ABC and TDF. Moreover, the limited availability and the high cost of boosted PIs force clinicians in RLS to recycle NNRTIs in the second-line regimen. Based on the resistance data from this study, we can assume that the effect of such a second-line regimen will be at the most temporary.

The small number of patients on a second-line regimen that were included and the limited time period between initiation of this regimen and the date of sampling, did not allow us to make conclusions about the efficacy of second-line regimens.

In conclusion, the results of this observational study show that effective first-line ART in clinical care centres with limited resources is feasible. However, the resistance data point out the danger of the absence of viral monitoring, with regard to the accumulation of resistance mutations.

Besides high quality adherence counselling, efforts are needed to guarantee a robust supply of drugs from different classes, as well as the worldwide availability of affordable and simple viral load and genotyping assays to ensure long-term success of global ART programs.

## Competing interests

The authors declare that they have no competing interests.

## Authors' contributions

KS designed the study, carried out molecular work and sequence analysis and prepared the manuscript. SL and CV assisted in designing the study and drafting the manuscript. KD and JR performed viral loads and genotyping. KM supervised the study in Mombasa. JW, JP and MT provided substantial intellectual content to the manuscript. All authors critically reviewed and approved the final manuscript.

## Supplementary Material

Additional file 1 **Table S1**. Characteristics of women and men at the time of study enrolment. Click here for file

Additional file 2 **Table S2**. Overview of resistance mutations detected in the RT gene of patients with a detectable viral load after more than 6 months of ART. Click here for file
